# Diversity, Distribution and Nature of Faunal Associations with Deep-Sea Pennatulacean Corals in the Northwest Atlantic

**DOI:** 10.1371/journal.pone.0111519

**Published:** 2014-11-04

**Authors:** Sandrine Baillon, Jean-François Hamel, Annie Mercier

**Affiliations:** 1 Department of Ocean Sciences, Memorial University, St. John's, Newfoundland and Labrador, Canada; 2 Society for the Exploration & Valuing of the Environment (SEVE), St. Philips, Newfoundland and Labrador, Canada; The Evergreen State College, United States of America

## Abstract

*Anthoptilum grandiflorum* and *Halipteris finmarchica* are two deep-sea corals (Octocorallia: Pennatulacea) common on soft bottoms in the North Atlantic where they are believed to act as biogenic habitat. The former also has a worldwide distribution. To assist conservation efforts, this study examines spatial and temporal patterns in the abundance, diversity, and nature of their faunal associates. A total of 14 species were found on *A. grandiflorum* and 6 species on *H. finmarchica* during a multi-year and multi-site sampling campaign in eastern Canada. Among those, 7 and 5 species, respectively, were attached to the sea pens and categorized as close associates or symbionts. Rarefaction analyses suggest that the most common associates of both sea pens have been sampled. Biodiversity associated with each sea pen is analyzed according to season, depth and region using either close associates or the broader collection of species. Associated biodiversity generally increases from northern to southern locations and does not vary with depth (∼100–1400 m). Seasonal patterns in *A. grandiflorum* show higher biodiversity during spring/summer due to the transient presence of early life stages of fishes and shrimps whereas it peaks in fall for *H. finmarchica*. Two distinct endoparasitic species of highly modified copepods (families Lamippidae and Corallovexiidae) commonly occur in the polyps of *A. grandiflorum* and *H. finmarchica*, and a commensal sea anemone frequently associates with *H. finmarchica*. Stable isotope analyses (δ^13^C and δ^15^N) reveal potential trophic interactions between the parasites and their hosts. Overall, the diversity of obligate/permanent associates of sea pens is moderate; however the presence of mobile/transient associates highlights an ecological role that has yet to be fully elucidated and supports their key contribution to the enhancement of biodiversity in the Northwest Atlantic.

## Introduction

Corals form one of the most complex biological habitats of the deep sea, offering a variety of microhabitats that serve as feeding, shelter, foraging and spawning sites to other species [1]–[4]. Deep-sea corals occur as unitary forms (i.e. composed of a single polyp) or colonial forms (i.e. composed of many polyps), and can be sparsely distributed or form fields, large thickets and even reefs that may stretch 300 m high and several kilometres wide [1], [2], [5]. A good understanding of deep-sea corals and their associated fauna, i.e. the organisms that live in or on the corals [1], is essential to evaluate the importance of these unique deep-sea ecosystems and to implement adequate measures for their conservation [6].

Studies of the associated fauna have shown that biodiversity around deep-sea corals can be comparable to that of tropical coral reefs and that main associates include crustaceans, molluscs, echinoderms, cnidarians, sponges, polychaetes and fishes [3], [7]–[9]. A review catalogued 983 invertebrate species associated with 74 species of deep-sea corals; 114 of the associates were characterized as symbionts (living in a close relationship with the coral host) of which 53% were parasites (detrimental to the host) and 47% were commensals (having no impact on the host) [7]. Deep-sea corals feed on zooplankton and phytodetritus, based on analyses of δ^13^C and δ^15^N [10], [11] as indicators of food sources and trophic levels, respectively [12]. However, to our knowledge, trophic relationships between deep-sea corals and their associated species have not been explicitly studied. So far, more studies have examined the fauna associated with hard corals than soft corals. We are aware of only one previous work on deep-sea octocorals in the Northwest Atlantic, which reported a total of 114 associates on 2 gorgonian species [1]. Additional information exists for soft corals (excluding Pennatulacea and Helioporacea) from other regions, with a total of 59 symbionts (83% listed as commensals and 17% as parasites) catalogued on 42 octocorals [13]. Sea pens (order Pennatulacea) are typically not afforded the attention of other deep-sea corals [7], [13] even though they are very common and have been identified as vulnerable organisms in both shallow and deep environments [4], [14]–[16]. Moreover, sea pens can be collected whole, allowing precise determination/quantification of faunal species living in, on or around them, which is not always the case with larger or more fragile branching corals (e.g. gorgonians) for which analyses of colony fragments is often the rule.

Sea pens can be considered “structural” species due to their extension above the seafloor [17] and have been suggested to create complex biohabitats [8]. However, so far no clear evidence has been provided to support their role as a biogenic habitat, although one study reported the presence of adult fish in large sea pen fields [18]. According to Etnoyer et al. [19], the majority of the species forming biogenic habitats exhibit complex morphology (e.g. branches) and a sufficient size to provide substrate or refuge for other species. Sea pens do not correspond to this definition but have nevertheless been shown to serve as biogenic substrate for different species [8], [20], [21] and to act as nursery habitat for fish larvae [3]. Moreover, sea pens can cover extensive areas in the deep sea, and are sometimes found in high densities [22], occurring on mud or sand flats, where they could provide an important structural biohabitat to other organisms [23] in relatively featureless environments.

Buhl-Mortensen et al. [8] noted that there seemed to be few species associated with sea pens, indicating that this observation was plausibly due to a lack of data, and only mentioned the association between the ophiuroid *Asteronyx loveni* and the sea pen *Funiculina quadrangularis* [8]. Other associates have been described, including a copepod parasite in *Anthoptilum grandiflorum* [24] in the Labrador Sea (1210 m depth) and a polychaete living between the polyps of *Funiculina quadrangularis* [20] along the Swedish coast (300 m depth). More associated species have been found in, on or around shallow-water sea pens, including different parasitic copepods on various host species [24]–[27], the gametophyte of an algae living inside the tissues of *Ptilosarcus gurneyi* [28], and the hydrozoan *Eudendrium ramosum* on *Virgularia mirabilis* [21]. At least 5 symbionts were reported on *Ptilosarcus gurneyi* [29], and a porcellanid crab was found between the leaves of *Pteroeides esperi* [30].

Pennatulacean corals are common on the continental slope of eastern Canada, where 16 species have been inventoried [4], [31]. The present study focuses on two of the most common ones: *Anthoptilum grandiflorum* (Anthoptilidae) and *Halipteris finmarchica* (Halipteridae) which were recently found to act as essential larval fish habitat [3]. *A. grandiflorum* exhibits a cosmopolitan distribution, with confirmed occurrence in the North and South Atlantic, North and South Pacific, Indian and Antarctic Oceans [32] while *H. finmarchica* is restricted to the North Atlantic [33]. Both species are present from 100 to >2000 m [22]. The main goal of this study was to better define their role and importance as biogenic substrate or habitat with the following objectives: (1) determine the diversity and abundance of their associated species, with an emphasis on spatial and temporal patterns; (2) characterize the dominant symbiotic relationships; and (3) elucidate trophic interactions between the most common associates and their hosts.

## Materials and Methods

### Collection

**Figure 1:**
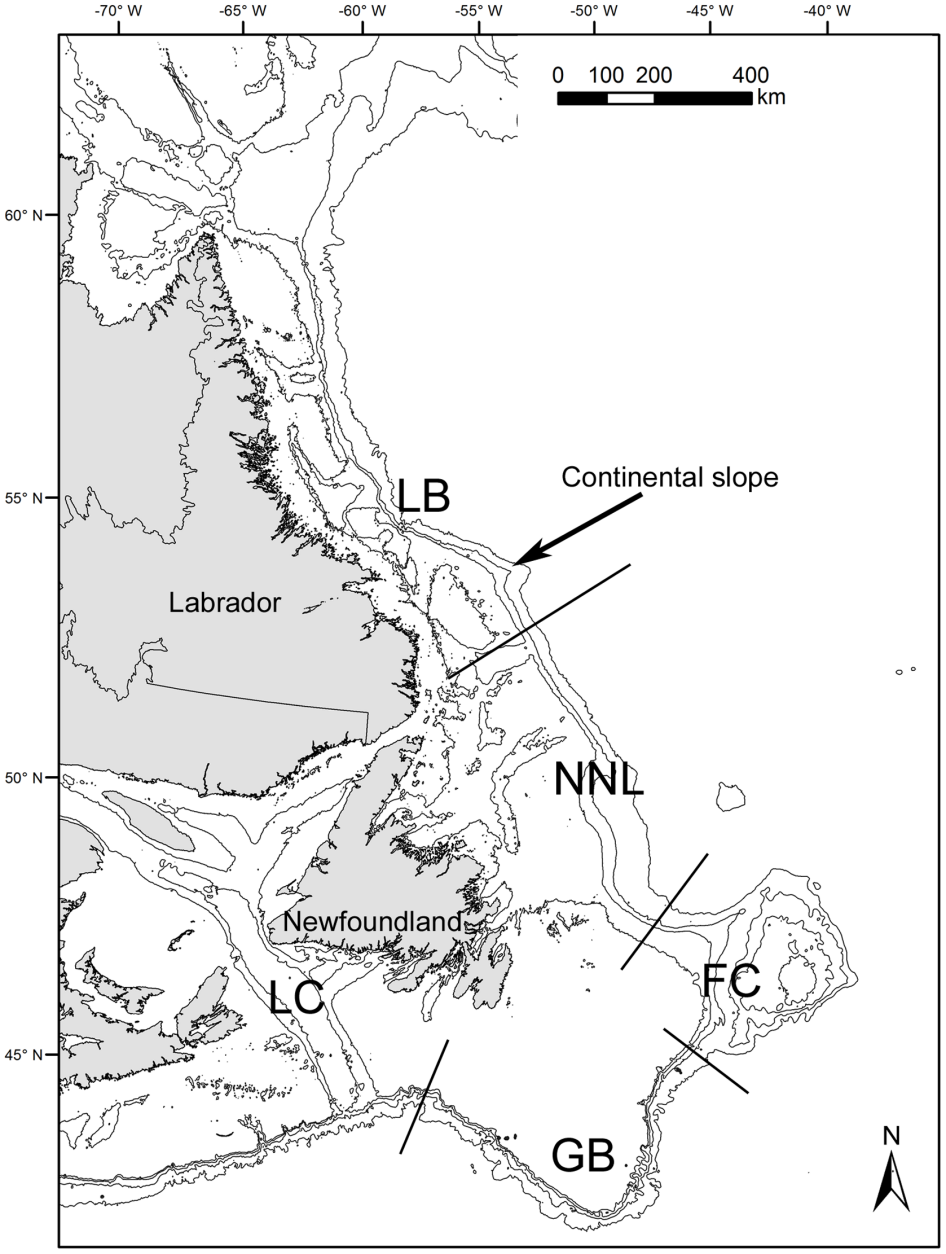
Map showing the five geographic regions where colonies of the sea pens *Anthoptilum grandiflorum* and *Halipteris finmarchica* were collected along the continental slope. LC: Laurentian Channel, GB: Grand Banks, FC: Flemish Cap, NNL: North Newfoundland, LB: Labrador.

Samples of *Anthoptilum grandiflorum* (from 98–1347 m) and *Halipteris finmarchica* (from 256–1333 m) were obtained in 2006 and 2007 as by-catch from annual research surveys (Multispecies Surveys and Northern Shrimp Research Surveys), and the At-Sea Observer Program, along the continental slope of eastern Canada (Fig. 1, Tables S1 and S2) which were all led by Fisheries and Oceans Canada (DFO). The DFO surveys followed a stratified random sampling design with a Campellen 1800 trawl towed for 15 minutes on approximately 1.4 km (gear opened and closed at depth). For more information on the At-Sea Observer Program see Wareham et al. [34]. The sampling area can be divided into 5 regions: Laurentian Channel (LC), Grand Banks (GB), Flemish Cap (FC), North Newfoundland (NNL) and Labrador (LB, Fig. 1, Table 1). Additional samples collected in April and May of 2009 and 2010 were used to determine the consistent presence of some associated suspected to be particularly abundant during the spring months. Colonies of *A. grandiflorum* and of *H. finmarchica* were frozen at −20°C on board the vessels.

**Table 1:** Number of colonies sampled in the different geographic regions.

	LC	GB	FC	NNL	LB
***Anthoptilum grandiflorum***	34	35	56	12	31
***Halipteris finmarchica***	11	33	25	1	18

LC: Laurentian Channel, GB: Grand Banks, FC: Flemish Cape, NNL: North Newfoundland, LB: Labrador.

### Processing of Samples

Colonies to be analysed were selected haphazardly among all samples from a given site. When less than three colonies were sampled at a site, all the colonies were analysed. When more than three colonies were available, a minimum of three colonies were analysed, more if needed, in order to reach a minimum of 20% of the colonies sampled at each site. Few exceptions occurred when samples were unavailable or damaged. Overall, samples of *A. grandiflorum* examined included 185 colonies (measuring 15–83.9 cm) in 2006–2007 (Table S1) and 60 colonies (19.8–76.8 cm) in 2009–2010 (Table S2). Samples of *H. finmarchica* consisted of 92 colonies (17.2–148.6 cm) in 2006–2007 (Table S1) and 12 colonies (15.8–94.0 cm) in 2009–2010 (Table S2). Colonies were thawed in filtered seawater before measuring colony length (from the peduncle to the tip of the sea pen), polyp diameter (n = 10) and density in the three rachis sections, coined lower, middle and upper section as in previous studies on sea pens [35]–[37]. Colonies were subsequently inspected under a stereomicroscope (Nikon SMZ1500) coupled to a digital camera (Nikon DXM1200F) to isolate and identify associated species. The position of each associate along the central axis was recorded (peduncle, lower, middle and upper sections of the rachis). After extraction from the sea pens, samples of associated species were preserved in 100% ethanol for DNA analyses or dried for 48 h at 60°C for isotopic analyses.

### Identification of the Associated Species

While there is no explicit or universal definition of faunal associates or associated species, the terms typically refer to species that find living space, shelter and/or food in or around a given substrate, habitat or species. Here, they were divided into three categories: (1) endobionts (living inside the tissues of the sea pen), (2) ectobionts (or epibionts, living attached to the surface of the sea pen) and (3) free-living. The latter were found unattached to the sea pen but trapped between the polyps, evoking a close association at the moment of sampling. Whenever there was doubt that a specimen might be a by-catch species, it was omitted from the analysis. It is important to note that free-living associates may be lost during sampling, leading to an underestimation of their importance. Studies have sometimes considered only the associates living inside or attached to the corals [1]. Therefore, analyses were conducted on all three categories (all associates) as well as on categories 1 and 2 only (close associates/symbionts). Associated species were grouped according to their morphology and identified to the lowest possible taxonomic level. For the dominant associates, measures of length (e.g. copepod) or basal diameter (e.g. sea anemone) were recorded.

A total of 93 samples of associates were processed by the Canadian Centre for DNA Barcoding (University of Guelph, Canada) for genetic identification. They were analyzed using standard polymerase chain reaction (PCR) and DNA sequencing protocols [38], [39]. Identifications were made by running the sequences against the BOLD and BLAST databases.

### Distribution of the Associated Species

The prevalence of associates (percentage of sea pen colonies harbouring a given species) was determined for pooled associates and for the three categories separately (endobiont, ectobiont, free-living; described above). The mean yield (MY) was defined as the mean number of associates per colony (ind colony^−1^) considering all sea pens examined, and the mean exact yield (MEY) was defined as the mean number of associates colony^−1^ considering only sea pens harbouring this associated species. Both measures were extrapolated to obtain total yields for the associates (MYtot and MEYtot), both overall and within each category of associate. The MY for a site (site mean yield [SMY] or site mean exact yield [SMEY]) was defined as the number of associates found in that site divided by the number of sea pen colonies examined for that site (as individuals colony^−1^). All parameters, i.e. prevalence, MY, MEY were also separately determined for the most common (major) associated species.

### Specificity of the Lamippidae and Corallovexiidae

Complementary data were obtained from histological sections of polyps of *A. grandiflorum* colonies infested by *L. bouligandi* that were preserved in 4% formaldehyde (n = 12). Polyp samples were prepared using standard histology protocols [40]. They were dehydrated in an ethanol series (70–100%), embedded in paraffin, sectioned (6–10 µm) and stained with haematoxylin and eosin. They were examined under a light microscope (Nikon Eclipse 80i) coupled to a digital camera (Nikon DXM1200F) and analyzed using the imaging software Simple PCI (v. 6.0).

To determine the effect of *Lamippe bouligandi* on the fecundity of *A. grandiflorum*, the density and Feret diameter of oocytes were determined in 5 polyps harbouring a copepod and 5 polyps without copepods sampled in a given colony. The measures were limited to the upper section of the colony to avoid the variation of fecundity along the rachis (increase of the fecundity from the lower to the upper section [37]).

### Trophic Interactions

Due to putative regional variations in carbon and nitrogen signatures of pennatulaceans [10], only samples from the Laurentian Channel sampled in 2007 were used for isotopic analysis; this location/date yielded several colonies with enough copepods to allow comparisons. Analyses of stable isotopes were conducted according to Sherwood et al. [10] on 16 samples of associates (2 *L. bouligandi*, 3 undescribed Corallovexiidae and 5 *S. nexilis*) and on their hosts (2 *A. grandiflorum* and 4 *H. finmarchica*). Briefly, dried samples were ground to powder and treated with 5% (v/v) HCl to remove carbonates, then rinsed three times with de-ionised water and dried again for 24 h at 60°C. Between 0.6 and 2.3 mg of sample was placed into 10×10 mm ultralight Sn capsules. Due to the small size of the copepods, specimens from a given colony were pooled to obtain the minimum weight necessary. The analyses were carried out using a Carlo Erba 1500 elemental analyser connected via a ConFlo-II interface to a FinniganTM MAT 252 isotope ratio mass spectrometer in the Department of Earth Sciences at Memorial University. The carbon and nitrogen isotopic values are provided using the standard δ-notation: δX  =  [(R_sample_/R_standard_)−1]×10^3^, where X corresponds to ^13^C or ^15^N and R is ^13^C/^12^C and ^15^N/^14^N, respectively.

As per Sherwood et al. [10] a proxy for particulate organic matter (POM) was used in the form of sedimentary organic matter (SOM) from the LC sampled at 268–531 m between October and December 1990 [41]. Data for pelagic and benthic invertebrates were not available for LC. However, previous data from offshore NNL were used [42] including amphipods and euphasiids for the pelagic invertebrates, and shrimps (*Pandalus borealis* and *Pasiphae multidentata*) and snow crab for benthic invertebrates to situate the sea pens in the food web.

Trophic level (TL) was estimated from the δ^15^N values using the following equation [43]: TL_consumer_  =  [(δ^15^N_consumer_−δ^15^N_base_)/Δδ^15^N]+TL_base_ where δ^15^N_consumer_ corresponds to the δ^15^N of the taxa considered, while δ^15^N_base_ and TL_base_ correspond to the value of the baseline of the trophic web considered, and Δδ^15^N is the trophic fractionation for δ^15^N (average 3.8‰ for polar and deep-sea studies [44]). Here, the base value was determined as per Gale et al. [45] using zooplankton as the primary consumer (TL_base_  = 2.3, δ^15^N_base_  = 9).

In addition, gastro-vascular contents of the sea anemone *Stephanauge nexilis* (an associate of *H. finmarchica*, see results) were extracted and preserved in 100% ethanol for DNA analyses. Eight samples were processed for DNA identification as outlined above.

### Data Analysis

Rarefaction curves [46] were used to compare species richness of faunal associates between sea pen host species using BioDiversity Pro software (Natural History Museum, London/Scottish Association of Marine Sciences). Rarefaction analysis allows an estimation of the number of species expected (E_(Sn)_) for a specific number of individuals observed (n) removing the influence of the sample effort [47]. The evenness (or equitability, indicating whether or not species are represented by a similar number of individuals) of the assemblage of species was determined for both sea pens using the Shannon–Wiener diversity index: 
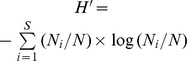
 [47] where S is the total number of taxa, N the total number of individuals, N_i_ the number of individuals of the i^th^ taxa. Biodiversity (rarefaction curve, expected number of species and the Shannon–Wiener diversity) was determined separately for all associates and for close associates (endobionts and ectobionts only).

Principal component analyses (PCA) were used to determine the influence of season and region on the species distribution at the studied sites. Data were pooled per site and a log(x+1) transformation was applied to the faunal abundance values [47]. This transformation allows the consideration of both the most abundant and rarer species. The general repartition of the associated species, their diversity and the repartition of the most common associates were analysed according to sea pen colony length, colony section, depth, region (Fig. 1; Laurentian Channel, Grand Banks, Flemish Cape, North Newfoundland, Labrador) and season. Additionally, sea pen morphometry (polyp density and polyp diameter) was used to analyze the fine scale distribution of the most common associated species. According to the parameter considered, linear regression and one-way ANOVA or t-test were used, after verifying assumptions of normality and homogeneity of variances. Post-hoc pairwise analysis (Student-Newman test) was conducted as appropriate. When assumptions were not met even after transforming the data, Spearman correlation and Kruskal-Wallis or Mann-Whitney tests were used, followed by Dunn's tests as appropriate. The number and distribution of ectobionts and free-living associates among seasons, depths and regions precluded the statistical analysis for these associates alone. Therefore, the analyses of seasonal, bathymetric and regional variations were carried out using MEYtot and biodiversity index. Due to the sample size, analysis of the influence of depth on the yield was carried out only when more than 10 colonies with associated species were sampled in the same region for a specific season. Therefore analyses were limited for *A. grandiflorum* to LC-spring (n = 28), FC-fall/winter (n = 39), GB-spring (n = 12), GB-fall (n = 24) and LB-summer (n = 20); while only GB-fall (n = 11) was used for *H. finmarchica*. For the influence of depth on biodiversity, all data irrespective of region and season were used and data were pooled per range of depth (100-m interval) to determine E_(S15)_. Comparison of biodiversity between seasons (fall vs. spring) was done on samples from GB and LB for *A. grandiflorum* and GB for *H. finmarchica*. Due to the difference in the number of associates found in the different regions/seasons, different expected number of species were used (E_(S170)_, E_(S150)_, E_(S120)_ and E_(S20)_, respectively).

## Results

### Species Identification and Diversity

**Figure 2:**
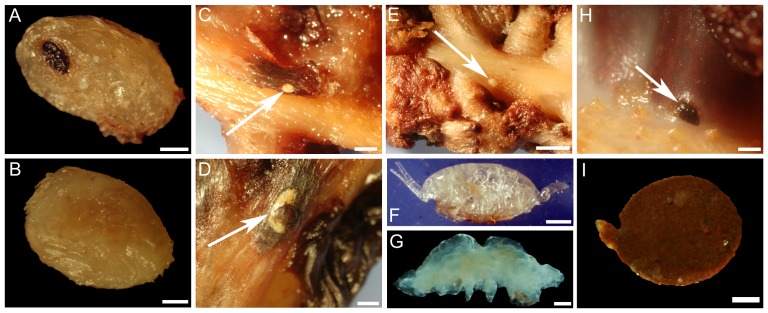
Associates of *Anthoptilum grandiflorum*: (A) decapod larva, (B) unidentified sp. 3, (C) unidentified sp. 1, (D) unidentified sp. 4, (E) unidentified sp. 2, (F) unidentified copepod. Unidentified sp. 1 to 4 correspond to potential egg mass. Associates of *Halipteris finmarchica*: (G) unidentified Lamippidae, (H and I) unidentified sp. 7. Scale bar in A = 200 µm, B, F and H = 500 µm, C and D = 2 mm, E = 4 mm, I = 100 µm. Species numbers linked to Table 2.

**Figure 3:**
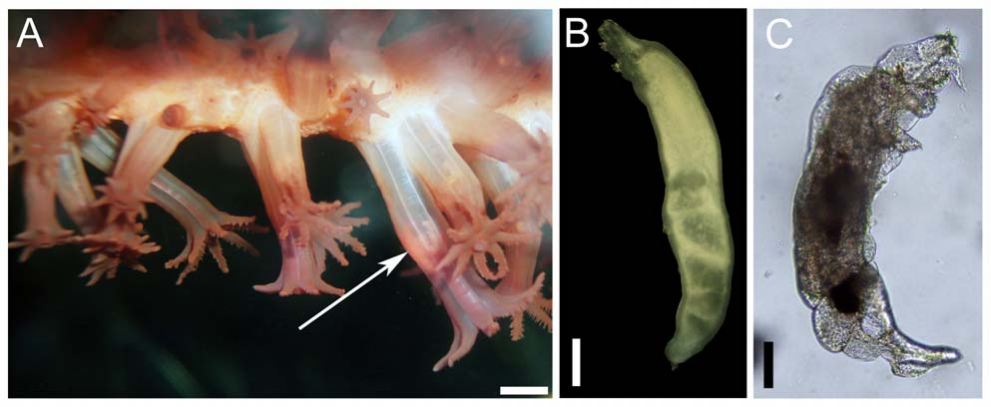
*Lamippe bouligandi*, a parasitic copepod living inside the polyps of *Anthoptilum grandiflorum*: (A) in situ view of the copepod (arrow) through the transparent polyp wall, (B) a female, (C) a male. Scale bar in A = 1 mm, in B = 500 µm and in D = 100 µm.

**Figure 4:**
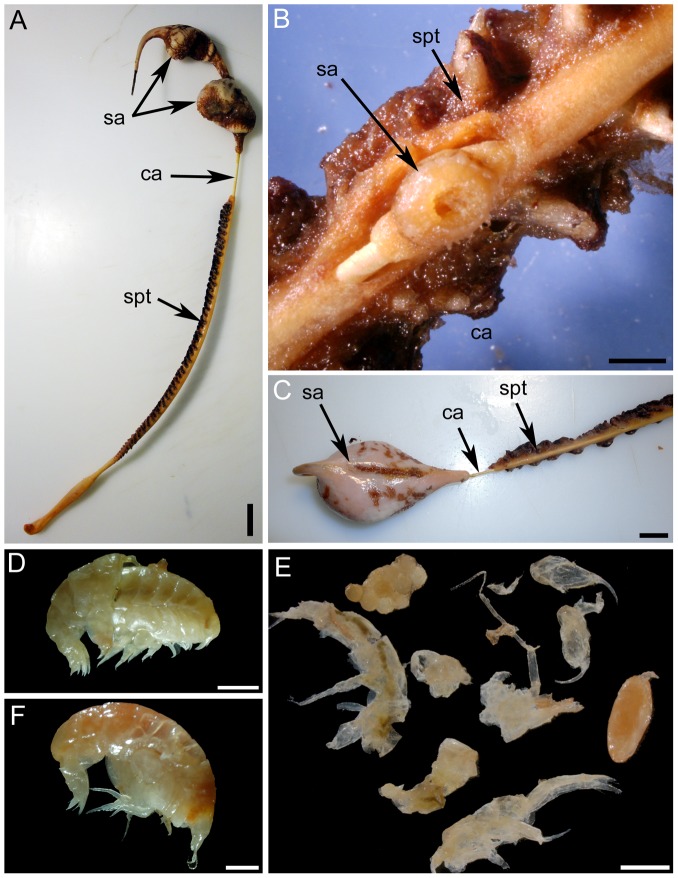
The sea anemone *Stephanauge nexilis* using the central axis of *Halipteris finmarchica* as a substrate: (A) general view of a colony of *H. finmarchica* harbouring two sea anemones in the upper section, (B) a small sea anemone surrounded by sea pen tissues, (C) dorsal view of the sea anemone found on the upper section of the sea pen colony. Gastro-vascular contents were found: (D and F) amphipod, (E) mix of prey including amphipods, halocyprids, egg mass, and unidentified food item extracted from one sea anemone. sa: sea anemone, ca: central axis, spt: sea pen soft tissues. Scale bar in A = 2 cm, in B and D = 2 mm and C and F = 1 cm, E = 1 mm.

**Figure 5:**
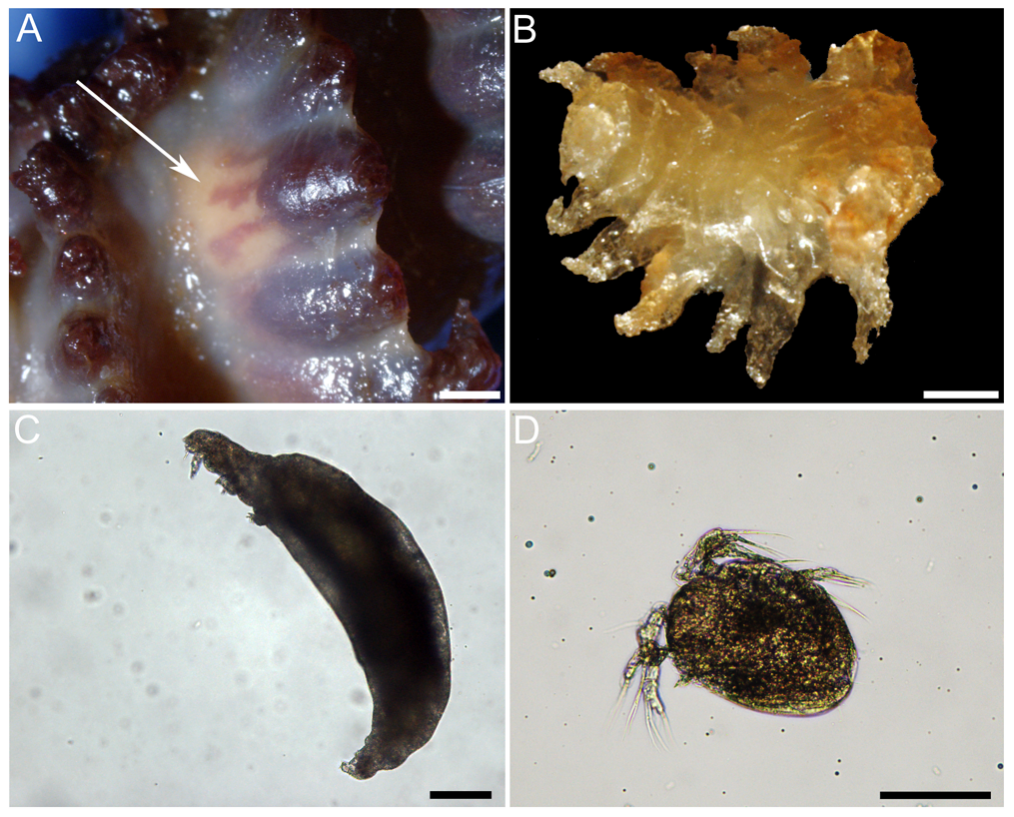
Undescribed copepod species belonging to Corallovexiidae living inside the polyps of *Halipteris finmarchica*: (A) row of polyps including a polyp infested with a copepod (arrow), (B) female copepod, (C) male copepod and (D) nauplius larvae. Scale in A = 1 mm, in B = 500 µm, in C and D = 100 µm.

A total of 1647 individuals belonging to 14 species (7 scored as close associates or symbionts) were found on the 175 colonies of *A. grandiflorum* examined and a total of 189 individuals belonging to 6 species (5 close associates) occurred on the 43 colonies of *H. finmarchica* (Table 2, Fig. 2, 3, 4 and 5). Seven species associated with *A. grandiflorum* were classified as free-living, 1 as ectobiont and 6 as endobionts, whereas 1 free-living associate, 2 ectobionts and 3 endobionts were found on *H. finmarchica*. On the 93 samples prepared for genetic identification, 52.7% were successfully sequenced. Partial COI sequences with all meta-data are registered in the Barcode of Life Data Systems [48], project SBDSC, and deposited in GenBank (Table S3). This analysis allowed identification down to species for fish and shrimp larvae. While no precise identification was obtained for the other specimens, higher taxonomic levels were determined.

**Table 2:** Species found on the sea pens *Anthoptilum grandiflorum* and *Halipteris finmarchica* during the present study (2006–2007).

Species	Number of individuals	Prevalence (%)	Type of association	Life stage	Link to pictures
**On ** ***Anthoptilum grandiflorum***					
Actinopterygii					
Scorpaeniformes					
*Sebastes* spp.	150	17.1	Free-living	Larvae	[3]
Myctophiformes					
*Benthosema glaciale*	1	0.4	Free-living	Larvae	[3]
Perciformes					
*Lycodes esmarkii*	1	0.4	Ectobiont	Egg	[3]
**Crustacea**					
Copepoda					
*Lamippe bouligandi*	1458	66.2	Endobiont	Adult	Fig. 4
Unidentified Copepoda	4	1.7	Free-living	Adult	Fig. 2F
Decapoda*					
*Acanthephyra pelagica*	2	0.4	Free-living	Larvae	Fig. 2A
*Pandalus montagui*	3	0.9	Free-living	Larvae	Fig. 2A
Unidentified Decapoda	7	1.7	Free-living	Larvae	Fig. 2A
Amphipoda					
Unidentified Amphipoda	3	1.3	Free-living	Adult	—
**Nematoda**					
Unidentified Nematoda	2	0.4	Free-living	Adult	—
**Unidentified species**					
Unidentified sp. 1	6	2.6	Endobiont	Egg	Fig. 2C
Unidentified sp. 2	2	0.9	Endobiont	Egg	Fig. 2E
Unidentified sp. 3	6	2.1	Endobiont	Egg	Fig. 2B
Unidentified sp. 4	1	0.4	Endobiont	Egg	Fig. 2D
Unidentified sp. 5	1	0.4	Endobiont	?	—
**On ** ***Halipteris finmarchica***					
**Actinopterygii**					
Scorpaeniformes					
*Sebastes* spp.	17	4.3	Free-living	Larvae	[3]
**Cnidaria**					
Actinaria					
*Stephanauge nexilis*	28	16.0	Ectobiont	Adult	Fig. 5
Hydrozoa					
Unidentified Hydrozoa	1	1.1	Ectobiont	Adult	—
**Crustacea**					
Copepoda					
Undescribed Corallovexiidae	112	29.8	Endobiont	Adult	Fig. 6
Unidentified Lamippidae	7	7.5	Endobiont	Adult	Fig. 2G
**Unidentified species**					
Unidentified sp 7	10	4.3	Endobiont	?	Fig. 2H, I

* 6 more larvae were found in April 2009 with a third species identified as *Pasiphaea multidentata*.

The free-living species included fish larvae (*Sebastes* spp. and *Benthosema glaciale* [3]), shrimp larvae (*Acanthephyra pelagica*, *Pandalus montagui*), amphipods, copepods and nematodes. The ectobionts included one occurrence of one egg of the fish *Lycodes esmarkii* attached to the tissues of one colony of *A. grandiflorum* [3], several sea anemones *Stephanauge nexilis* and a hydrozoan colony found on the naked upper section (exposed skeleton) of colonies of *H. finmarchica*. Finally the endobionts included parasitic copepods (*Lamippe bouligandi* on *A. grandiflorum*, an undescribed Corallovexiidae and an unidentified Lamippidae both found on *H. finmarchica*) and 6 unidentified species (including 4 putative egg masses on *A. grandiflorum*; Table 2).

### Analysis of Close Associates

When only the close associates were considered (endobionts and ectobionts), the values of E_(S150)_ and evenness were lower for *A. grandiflorum* than *H. finmarchica* (E_(S150)_: 2.45 and 4.00, H′: 0.07 and 0.84, respectively). Rarefaction curves did not reach the asymptote. Overall, 97.9% of the individuals found on the two sea pens belonged to 3 species. The most common (89.3% of the associates) occurred on *A. grandiflorum* and was identified as *Lamippe bouligandi*, a parasitic copepod living inside the tissues of the polyp column (Fig. 3A). The next two most common species were found on *H. finmarchica*: a sea anemone (representing 1.7% of the associates) found attached to the central axis, showing 96% DNA similarity with Hormathiidae and identified as *Stephanauge nexilis* (Fig. 4A), and a parasitic copepod (6.8% of the associates; Fig. 5A) living inside the polyp, in the space typically hosting reproductive cells. The latter was identified as a copepod from the family Corallovexiidae based on the presence of nauplii (characteristic of crustacean) and its general morphology. The parasitic copepod found in *H. finmarchica* presents lateral extensions (5 or 6 pairs of pereionites) consistent with the Corallovexiidae described by Stock [49]. The male of the undescribed Corallovexiidae, which was always found close to the female, surrounded by eggs/nauplius, differs from previous descriptions. However, only 10 species have so far been described, and it is likely that variation in the shape of males exist. Finally, a genetic similarity of ∼85.5% was obtained between the undescribed Corallovexiidae and *L. bouligandi* (family: Lamippidae) suggesting that the two species belong to different families. Given the localisation of these copepods in their hosts, they were considered endobionts.

Principal component analysis (PCA) on the close associates of *A. grandiflorum* revealed that the copepod *L. bouligandi* was the main contributor to the first principal component (PC1: 94.0%) and the unidentified sp. 1 to the second principal component (PC2: 4.0%). For *H. finmarchica* the main contributor to the first principal component was the undescribed Corallovexiidae (PC1: 65.5%) and the sea anemone *S. nexilis* for the second component (PC2: 21.4%). No clear grouping was visible on the PCAs for any sea pen.

Seasonal analyses showed a higher diversity of species associates with *A. grandiflorum* in spring/summer (E_(*S*200)_  = 3.25) than in fall (E_(*S*200)_  = 1.66) while the MEYtot showed no variation among seasons (H = 4.04, df  = 3, P = 0.258). The opposite trend was observed for *H. finmarchica* with a lower diversity in spring/summer (E_(*S*40)_  = 2.3) compared to fall (E_(*S*40)_  = 4). However, the MEYtot showed no seasonal variation (H = 2.71, df = 2, P = 0.258).

**Figure 6:**
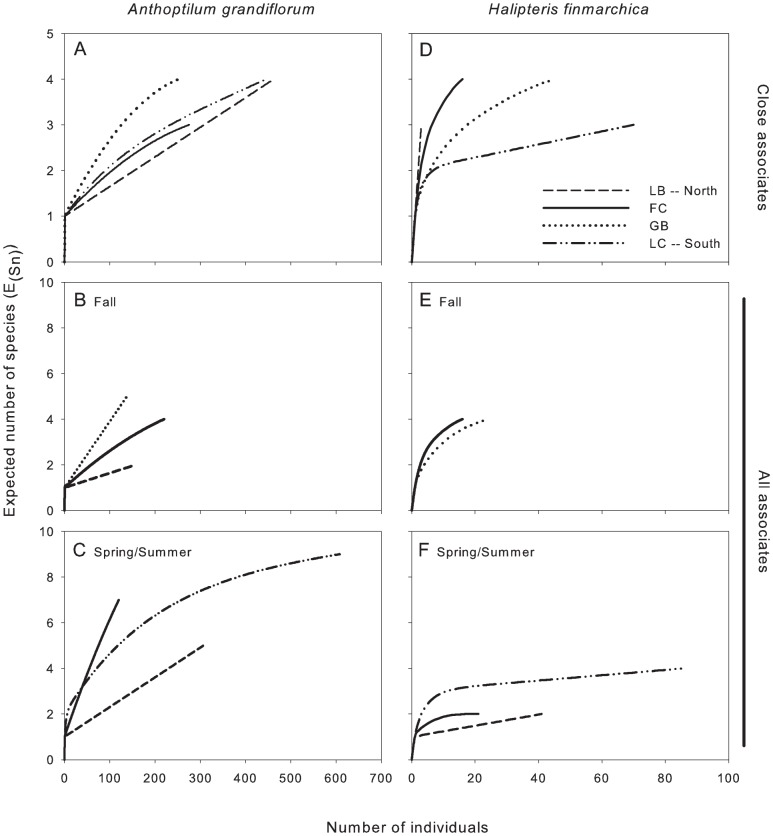
Rarefaction curves for *Anthoptilum grandiflorum* (left panels) and *Halipteris finmarchica* (right panels), based on close associates only (top two panels) or all associates in spring and fall (bottom four panels). (A) Close associates of *A. grandiflorum*; (B) all the associated fauna of *A. grandiflorum* in the fall and (C) in spring/summer. (D) Close associates of *H. finmarchica*; (E) all the associated fauna of *H. finmarchica* in the fall and (F) in spring/summer. LC: Laurentian Channel, GB: Grand Banks, FC: Flemish Cap, NNL: North Newfoundland, LB: Labrador.

Regional analyses showed different biodiversity associated with *A. grandiflorum* among regions (Fig. 6A); however, no pattern was visible and no variation of the MEYtot was detected (H = 8.95, df  = 4, P = 0.062). A general southward decrease emerged for the biodiversity associated with *H. finmarchica* among regions (Fig. 6D) while no variation of the MEYtot occurred (H = 1.65, df  = 3, P = 0.648).

There was no influence of depth on the biodiversity associated with either sea pen species (*A. grandiflorum*: r^2^  = .11, F_(1,6)_  = 0.60, P = 0.474, log-transformed data; *H. finmarchica*: r^2^ = 0.12, F_(1,4)_  = 0.42, P = 0.563) or their MEYtot (*A. grandiflorum*: r*_s_* = −0.06, P = 0.429, log-transformed data; *H. finmarchica*: r*_s_* = −0.21, P = 0.187).

### Analysis of All Associates

Values of E_(S170)_ when all species found considered were lower for *A. grandiflorum* (∼5 expected species) than *H. finmarchica* (∼6 expected species). The rarefaction curve for *A. grandiflorum* did not reach an asymptote while the curve for *H. finmarchica* showed a steeper increase of the number of species towards an asymptote. However, when the rarest species (with only one observation) were removed, the rarefaction curve of both species reached an asymptote. Evenness was lower for *A. grandiflorum* (H′ = 0.44) than for *H. finmarchica* (H′ = 1.12).

Larvae of redfish, *Sebastes* spp., were the fourth most common species found during this study (representing 9.3% of associates); they were present on both sea pens (for more details see Baillon et al. [3]). In addition to fish larvae, 12 shrimp larvae were found in April 2006 and April 2007 on *A. grandiflorum*; they were identified as *Acanthephyra pelagica* (DNA: 99% certainty) and *Pandalus montagui* (DNA: 100% certainty). Six shrimp larvae were also found on four colonies of *A. grandiflorum* in April 2009, one of them identified as *Pasiphaea multidentata* (DNA: 100% certainty).

**Figure 7:**
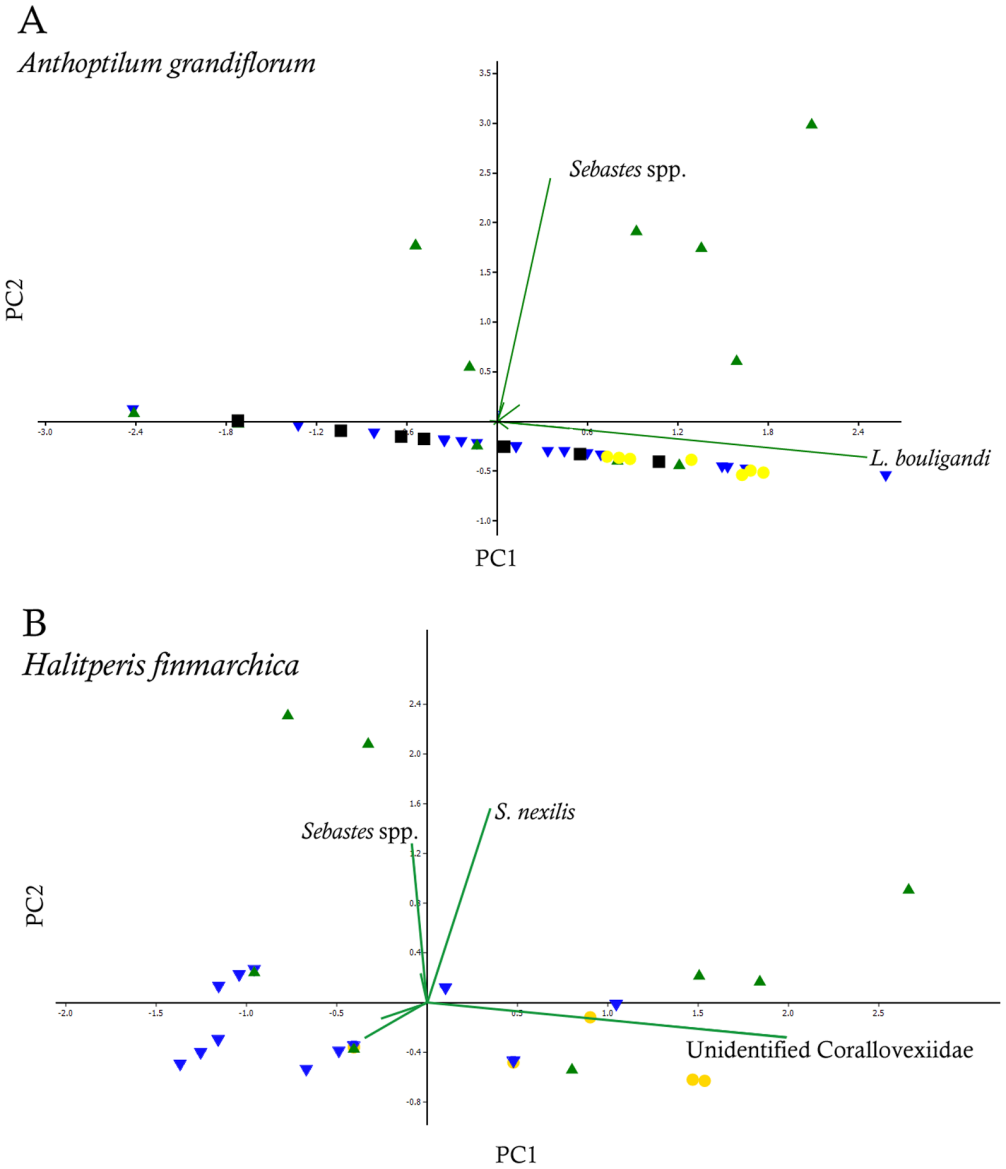
Principal component analyses and biplots based on the associated species of *Anthoptilum grandiflorum* (A, B) and *Halipteris finmarchica* (C, D). Green up triangle: spring; yellow circle: summer; blue down triangle: fall; black square: winter.

Principal component analysis (PCA) on the associated species of *A. grandiflorum* revealed that the copepod *L. bouligandi* was the main contributor to the first principal component (PC1: 69.1%) and the fish larvae to the second principal component (PC2: 22.5%). Two groupings were visible (Fig. 7A) corresponding, for the first, to the colonies harbouring fish larvae (April-May in the LC region, Fig. 1) and, for the second group, to all other samples in various regions/months. PCA on the associated species of *H. finmarchica* showed that the undescribed Corallovexiidae was the main contributor to the first principal component (PC1: 56.0%) and fish larvae and the sea anemone *S. nexilis* to the second principal component (PC2: 24.1%). However, no specific groupings emerged (Fig. 7B). Therefore, to account for the influence of fish larvae on the repartition of the study sites, the remaining analyses were conducted considering both regions and seasons (spring/summer vs. fall/winter).

Seasonal analyses inside specific regions showed that the diversity of species associated with *A. grandiflorum* was higher in spring/summer than in fall in GB (E_(*S*120)spring_  = 7.0> E_(*S*120)fall_  = 4.5) and LB (E_(*S*150)spring_  = 3.0> E_(*S*150)fall_  = 2.0). However, the MEYtot did not show any significant seasonal variations at any site (GB: U = 139.0, P = 0.596; LB: U = 94.5, P = 0.826). The associates of *H. finmarchica* showed a lower diversity in spring than fall in GB (E_(*S*20)spring_  = 2.0<E_(*S*20)fall_  = 3.8) but no significant difference in MEYtot was observed in GB (U = 27.0, P = 0.565).

Regional analyses within the various seasons revealed that the associated biodiversity of *A. grandiflorum* exhibited a general northward decrease in fall and spring/summer (Fig. 6B and C) while the MEYtot showed no significant variation among regions (spring/summer: F_(2,91)_  = 2.82, P = 0.065; fall: H = 4.72, df  = 3, P = 0.193; log-transformed data). In fall, *H. finmarchica* showed the same biodiversity of associates in FC and GB (E_(S15)_  = 3.94 and 3.48, respectively; Fig. 6E) as well as the same MEYtot (U = 37.0, P = 0.925), while in summer colonies showed a higher biodiversity of associates in LC than FC and LB (Fig. 6F) but no regional differences in MEYtot (F_(2,22)_  = 1.49, P = 0.247; log-transformed data).

No significant influence of depth was found on the biodiversity of associates for either sea pen host (*A. grandiflorum*: r^2^ = 0.21, F_(1,8)_  = 1.83, P = 0.218, *H. finmarchica*: r^2^ = 0.03, F_(1,5)_  = 0.14, P = 0.721). No bathymetric variation in MEYtot was found either, except a decrease of MEYtot with depth in GB during the fall for *A. grandiflorum* (Table 3).

**Table 3:** Influence of increasing depth on total mean yield (MEYtot) of all associates on colonies of *Anthoptilum grandiflorum* and *Halipteris finmarchica* in the different geographic regions (only sites with more than 10 colonies harbouring associated species were used).

Species	Region	Depth range (m)	n	Spring/summer	Fall/winter
***A. grandiflorum***	LC	301–488	28	r^2^ = 0.07, F_(1,27)_ = 1.81, P = 0.190	
	GB	98–603	13/24	r^2^ = 0.03, F_(1,11)_ = 0.34, P = 0.570	r^2^ = 0.41, F_(1,23)_ = 15.06, P<0.001
	FC	273–1208	39		r*_s_* = −0.06, P = 0.734
	LB	176–883	20	r^2^ = 0.02, F_(1,19)_ = 0.34, P = 0.569	
***H. finmarchica***	GB	579–1333	11		r^2^ = 0.01, F_(1,10)_ = 0.05, P = 0.825

LC: Laurentian Channel, GB: Grand Banks, FC: Flemish Cape, LB: Labrador, n: number of sea pen colonies analysed. Empty cells correspond to regions without enough data available for analyses.

### Species Distribution on the Hosts

All faunal associates were found on the rachis section of the host colonies. At least one of the associates was found on 75.9% of *A. grandiflorum* and 46.6% of *H. finmarchica* colonies. Across regions, prevalence proportion varied between 58.3% (NNL) and 96.8% (LB) for *A. grandiflorum* and between 23.8% (FC) and 90.0% (LC) for *H. finmarchica* (Table 4). For both species the endobionts were the most common (prevalence on *A. grandiflorum*  = 72.3%; on *H. finmarchica*  = 38.6%) across geographic regions. They were principally represented by *L. bouligandi* (98.9%) in *A. grandiflorum* and by the undescribed Corallovexiidae (87.5%) in *H. finmarchica*.

**Table 4:** Prevalence of associates on colonies of *Anthoptilum grandiflorum* and *Halipteris finmarchica* in the different geographic regions (as percent colonies harbouring them).

		All regions combined	LC	GB	FC	NNL	LB
***A. grandiflorum***	All associates	75.9	67.5	74.0	70.0	58.3	96.8
	Endobionts	72.3	52.9	72.2	70.0	58.3	96.8
	Ectobionts	0.6	0.0	0.0	3.3	0.0	0.0
	Free-living	26.0	52.9	8.3	0.0	0.0	16.1
***H. finmarchica***	All associates	44.7	90.9	44.7	23.8	—	55.6
	Endobionts	37.2	45.5	36.8	23.8	—	55.6
	Ectobionts	20.0	63.6	18.4	4.8	—	0.0
	Free-living	4.3	27.3	0.0	4.8	—	5.6

Data also shown separately for endobionts, ectobionts and free-living associates. LC: Laurentian Channel, GB: Grand Banks, FC: Flemish Cape, NNL: North Newfoundland, LB: Labrador.

The yield of associates (as MEY) on *A. grandiflorum* was significantly greater for endobionts (9.3±0.9 ind colony^−1^) than for ectobionts (1.0±0.0 ind colony^−1^) and for free-living species (3.2±0.7 ind colony^−1^; H = 42.83, df  = 2, P<0.001). No significant differences were found in the MEY of each category of associate on *H. finmarchica* (endobiont: 4.0±0.9 ind colony^−1^; ectobiont: 1.9±0.5 ind colony^−1^; free-living: 4.3±2.3 ind colony^−1^; H = 4.64, df  = 2, P = 0.099). Comparisons between the two sea pens showed that they harboured the same number of ectobionts (U = 30.0, P = 0.121) and free-living associates (U = 84.5, P = 0.401) whereas *A. grandiflorum* hosted a significantly higher number of endobionts than *H. finmarchica* (U = 1707.0, P<0.001).

Endobionts were present in all the sections of the rachis in both sea pen species. A significant increase of the endobiont MEY occurred from the lower to the upper section of *A. grandiflorum* colony (H = 95.50, df  = 2, P<0.001), while the endobionts in *H. finmarchica* showed a significantly higher MEY in the middle section than in the two other sections (middle> lower  =  upper; H = 12.39, df  = 2, P = 0.002). For both sea pens, when removing the most common associate (i.e. *L. bouligandi* and the undescribed Corallovexiidae), no significant differences were found among sections for other associates (*A. grandiflorum*: H = 3.10, df  = 2, P = 0.212; *H. finmarchica*: F_(2.6)_  = 1.5, P = 0.296). In *H. finmarchica*, the sea anemone *S. nexilis* and a hydrozoan (ectobionts) were always attached directly to the central axis in the upper section of the colonies. *A. grandiflorum* showed a significant increase of the MEYtot with colony length (r*_s_* = 0.16, P = 0.036) while no variation was noted for *H. finmarchica* (r*_s_* = 0.05, P = 0.735). Analyses per category of associate showed an increase of the MEY with colony length for free-living associates (r*_s_* = 0.36, P = 0.007) of *A. grandiflorum* while no variation occurred for other categories in either sea pen species.

### Relationship between Hosts and Dominant Associates

#### *Lamippe bouligandi* in *Anthoptilum grandiflorum*

A total of 1126 females and 23 males of the copepod *L. bouligandi* (MEYtot  = 9.4±0.9 copepods colony^−1^) were recorded from 118 colonies (15–84 cm) of *A. grandiflorum* (prevalence of 71.1%) from all five geographic regions under study. Eggs and nauplius larvae of *L. bouligandi* were found in association with 36 females (3.2%) in 18 sea pen colonies (10.8%) sampled year-round. Females mainly occurred singly in a polyp; whereas males were always paired with a female. The female copepods measured 5.06±0.07 mm (Fig. 3B) while the males were smaller at 1.39±0.17 mm (Fig. 3C). Two females occurred in the same polyp on 25 occasions (in 18 sea pen colonies) while larger groups of 3–4 females were found in only 4 polyps distributed on 3 colonies sampled year-round. No seasonal pattern emerged to explain the pairings/groupings. Infestation was between 0.1 and 19.1% of the polyps in an affected colony (i.e. 1–50 polyps). Overall, most (57.6%) of the colonies had less than 2% of polyps infested and only 3.4% of the colonies had>10% of polyps infested. An average of 44.0±4.7 white/yellowish oocytes were present in non-infested polyps and measured 429.1±11.7 µm. The infested polyps showed a significantly lower fecundity (19.6±5.1 oocytes polyp^−1^, representing a 45±6.9% decrease in relative fecundity) than the non-infested polyps (t = 3.51, df  = 8, P = 0.008), and they were translucent and significantly larger (520.5±18.8 µm, U = 7591.5, P<0.001).

No influence of colony length on the yield (MEY) of copepods was found (r*_s_* = 0.17, P = 0.057). A significant increase in the abundance of female copepods occurred from the lower to the upper section of the rachis (H = 77.71, df  = 2, P<0.001), with 60.3% of females occupying the upper section. Positive correlations were found between the abundance of copepod and both polyp density (r*_s_ = *0.34, P<0.001) and polyp diameter (r*_s_ = *0.31, P = 0.005). Infestation with *L. bouligandi* occurred at all sampling depths. No correlation of MEY with depth (r*_s_* = −0.16, P = 0.075) and no influence of season (H = 4.06, df  = 3, P = 0.255) were detected. However, significant regional differences in MEY were evidenced (H = 13.49, df  = 4, P = 0.009) between LB (16.07±0.95 copepods colony^−1^) and GB (5.04±0.95 copepods colony^−1^).

#### Undescribed Corallovexiidae in *Halipteris finmarchica*

A total of 112 females and 2 males copepods belonging to the Corallovexiidae (MEYtot  = 4.7±1.0 copepods colony^−1^) were recorded inside the polyps (Fig. 5A) of 28 colonies (21–132 cm) of *H. finmarchica* (prevalence of 29.8%) from all five geographic regions under study. When a male was found, it was always paired with a female. Females measured 4.52±0.51 mm (Fig. 5B) and males were smaller at 0.73±0.05 mm (Fig. 5C). Females occurred at the base of the polyp where reproductive cells typically grow (Fig. 5A). No oocytes or spermatocysts were observed in the infested polyps while the surrounding non-infested polyps harboured oocytes or spermatocysts. Overall, 61.6% of the female copepods were found in association with eggs/nauplii (Fig. 5D) at various times of the year. Contrarily to *L. bouligandi* in *A. grandiflorum*, a polyp never hosted more than one female Corallovexiidae. Infestation rates varied between 0.1 and 1.6% (1–20 infested polyps) in an affected colony with only five colonies (17%) harbouring more than 5 copepods.

MEY was not influenced by colony length (r^2^ = 0.07, F_1,22_  = 1.56, P = 0.225). The middle section of the rachis showed greater infestation than the upper and lower sections (H = 13.46, df  = 2, P<0.001), with 50% of the corallovexiids occurring there. This copepod was present at all depths sampled. Despite a significant decrease of the MEY with depth (r*_s_* = 0.52, P = 0.010), no clear threshold was detected; i.e. there was no significant difference among 100-m depth intervals (H = 7.24, df  = 7, P = 0.404). Comparison among seasons showed a higher MEY in spring than in fall (H = 7.98, df  = 2, P = 0.019). No significant regional differences were evidenced (F_2,20_  = 2.39, P = 0.117).

#### *Stephanauge nexilis* on *Halipteris finmarchica*

A total of 28 sea anemones *S. nexilis* were found attached to the central axis of *H. finmarchica*, usually in the upper section of the rachis that was devoid of soft tissues (Fig. 4A and C). However, three small individuals were found surrounded by polyps (Fig. 4B). Sea anemones had a basal diameter ranging from 0.4 to 9.9 cm (3.4±0.5 cm). Between 1 and 8 sea anemones (MEYtot  = 4.7±1.0 anemones colony^−1^) were found on 14 colonies of *H. finmarchica* (prevalence of 15.4%).

*Stephanauge nexilis* was present on *H. finmarchica* colonies from all sampling depths studied (366–1125 m) with no influence of depth on the MEY (r*_s_* = −038, P = 0.178). However, this association was restricted to the southern regions (85.7% in LC and GB, and 14.3% in FC). No significant seasonal difference in MEY was found (U = 12, P = 0.142).

#### Trophic Interactions between Hosts and Dominant Associates

**Figure 8:**
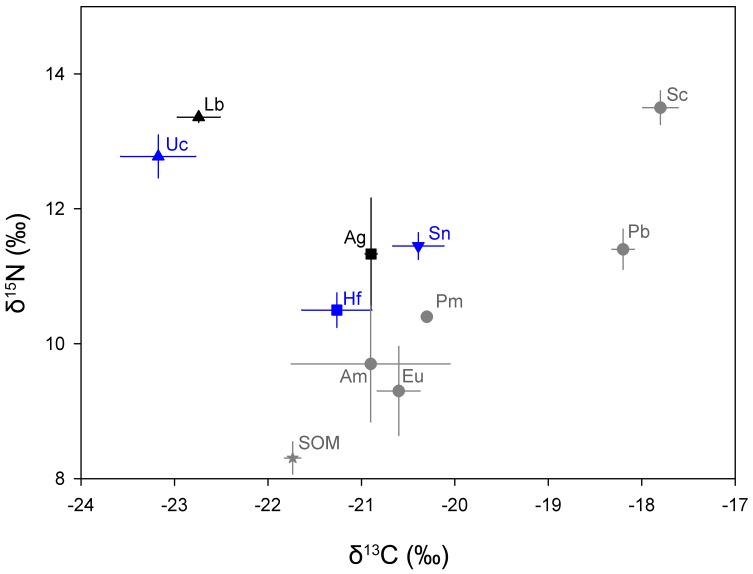
Stable isotope values (δ^15^N and δ^13^C) for sea pens (Ag: *Anthoptilum grandiflorum* and Hf: *Halipteris finmarchica*) and their associated species (Lb: *Lamippe bouligandi*, Sn: *Stephanauge nexilis* and Uc: undescribed Corallovexiidae). To locate and compare the signature of the sea pens, values for other invertebrates are shown, Am: Amphipods, Eu: Euphausiids, Pm: *Pasiphae multidentata*, Pb: *Pandalus borealis* and Sc: snow crab from Sherwood & Ross [42], as well as sedimentary organic matter (SOM) from Muzuka & Hillaire-Marcel [41]. Result shown as mean ± SE (n = 2–5). Black: *A. grandiflorum* and its associates, Blue: *H. finmarchica* and this associates, Grey: other invertebrates and SOM.

Analysis of isotopic ratios in tissues of the two sea pen species collected from LC showed they had similar δ^13^C and δ^15^N signatures (Table 5; δ^13^C: U = 3.5, P = 0.800; δ^15^N: U = 2.0, P = 0.533). No significant differences were detected between the sea anemone *S. nexilis* and its host *H. finmarchica* despite the fact that the sea anemone had a higher δ^13^C (∼1 ‰, t = −1.36, df  = 6, P = 0.224) and δ^15^N (∼1 ‰, t = −2.42, df  = 6, P = 0.052). Both sea pens and the sea anemone showed the same TL (Table 5). The two associated copepods had similar δ^13^C and δ^15^N signatures (Table 5; δ^13^C: t = −1.12, df  = 3, P = 0.344; δ^15^N: t = −1.40, df  = 3, P = 0.255). They had a significantly lower δ^13^C (∼2 ‰, F_(4,14)_  = 22.16, P<0.001) and a significantly higher δ^15^N (∼2 ‰, F_(4,14)_  = 10.12, P = 0.002) than their sea pen hosts (Fig. 8). On average, copepods were approximately half a trophic level (0.4–0.6) above their hosts.

**Table 5:** Carbon and nitrogen stable isotope signatures (δ^13^C and δ^15^N), and trophic level (TL) of *Anthoptilum grandiflorum* and *Halipteris finmarchica* and their dominant associates.

	n	δ^13^C (‰)	δ^15^N (‰)	TL
***Anthoptilum grandiflorum***	2	−20.9±0.1	11.3±0.8	3.0
***Lamippe bouligandi***	2	−22.7±0.2	13.4±0.1	3.4
***Halipteris finmarchica***	4	−21.3±0.4	10.5±0.3	2.7
***Stephanauge nexilis***	5	−20.4±0.3	11.5±0.2	2.9
**Undescribed Corallovexiidae**	3	−23.3±0.3	12.8±0.3	3.3

n: number of samples analysed (mean ± SE).

Gastro-vascular contents analysed in 8 of the sea anemones (28.5%) comprised small pelagic invertebrates: amphipods, copepods and halocyprids (based on DNA; Fig. 4D–F).

## Discussion

Different measures of biodiversity exist and its estimation depends on the number of species and the respective abundance of those species [50]. When considering only the close associates, biodiversity expressed as E_(S150)_ showed a higher diversity for *Halipteris finmarchica* than *Anthoptilum grandiflorum*. Both species exhibited a moderate number of associated species (see below) but additional associates still remain to be found based on the rarefaction curves. When all categories of associates were considered, E_(S170)_ was similar between faunal associates of *A. grandiflorum* and *H. finmarchica*; however, the rarefaction curves showed that increasing sample size would yield a greater numbers of associates for *A. grandiflorum* probably due to the higher number of free-living species found in association with this host (see below). When removing the rarest species (single occurrences), the rarefaction curves reached an asymptote, suggesting that the most common associates of both sea pens have been sampled. The Shannon-Weiner index ascribed more even abundances to the associates of *H. finmarchica* than to those of *A. grandiflorum*. Associates of *A. grandiflorum* are clearly dominated by one species, i.e. the copepod *Lamippe bouligandi*. Associates of *H. finmarchica* comprise two common species, an undescribed Corallovexiidae (Copepoda) and the sea anemone *Stephanauge nexilis*, resulting in a slightly more even distribution. Overall, specialized copepods emerge as the predominant associates of sea pens.

In general, measures of biodiversity associated with each sea pen species showed comparable patterns of variation with depth, region and season, irrespective of whether all or only close associates were considered, with a single exception outlined below. Variations in richness of faunal associates were not observed across depths in any of the analyses. A northward decrease was generally detected, except for the close associates of *H. finmarchica*, which showed a southward decrease. The northward decrease is in accordance with previous studies reporting a general decline of biodiversity with increasing latitude [51], [52]. Variation in primary productivity over large spatial scales has been proposed to generate this trend [53]. The fact that associated biodiversity showed different seasonal peaks for the two sea pens species is intriguing. The higher biodiversity in spring for *A. grandiflorum* may be explained by the presence of egg masses and early life stages of free-living species following spring reproductive events. However, no clear explanation emerges for the higher fall biodiversity associated with *H. finmarchica*.

Sea pen colonies studied here only yielded associated species on the rachis, and none on the peduncle. This is not unexpected since the peduncle is essentially buried in the sediment in both *A. grandiflorum* and *H. finmarchica*. However, a polychaete was recorded in association with the peduncle of sea pen colonies that had been maintained alive in the laboratory for a few weeks (including *A. grandiflorum* and *H. finmarchica*); the polychaete appears to be a new deep-sea species that feeds opportunistically on sea pen flesh [54]. An earlier report by Johnstone [29] described the presence of a parasitic copepod living in/on the half-buried peduncle of the shallow-water sea pen *Ptilosarcus guerneyi* from the North Pacific.

The number of associates identified in *A. grandiflorum* and *H. finmarchica* is similar to that reported in the shallow-water sea pen *P. guerneyi* [29] from Puget Sound (Northeast Pacific), suggesting that the biodiversity associated with pennatulacean octocorals may be consistent across regions and depths. It is apparently lower than that generally reported in deep-water branching corals, keeping in mind that comparison among taxonomic groups is often problematic, due to differences in methods and sampling effort. For example, 66 species have been found on seven partial colonies of the scleractinians *Madrepora oculata* and *Lophelia pertusa* sampled by trawl in the Mediterranean Sea [55]. As for octocorals in the order Gorgonacea (sea fans), 47 and 97 associated species have been found on 13 colonies/fragments of *Paragorgia arborea* and 45 colonies/fragments of *Primnoa resedaeformis*, respectively, that were either sampled by ROV or by trawl in the Northwest Atlantic [1]. The E_(S170)_ of *P. arborea* and *P. resedaeformis* is around 18 and 38 expected species, respectively [1], which is 3–6 times higher than E_(S170)_ in *A. grandiflorum* and *H. finmarchica* from the same geographic region. The difference in the diversity of associates likely results from the type of substrate/habitat offered by sea pens vs. sea fans, as well as from the inherently different biodiversity of their respective environments (soft vs. hard bottoms). Two different microhabitats occur in gorgonians: (1) living tissues in the young body parts of the colony and (2) exposed skeleton in the older body part of the colony [1]. The former harboured a lower biodiversity but the highest abundance of specialized associates. The greater biodiversity in the older/dead section is due to the capacity of sessile species to settle there, as also observed in dead sections of deep-sea scleractinians [56], [57]. The moderate biodiversity associated with sea pens might therefore be due to the less frequent availability of exposed skeleton for other species to colonize. The central axis of sea pens is formed of collagen and calcite [58], and provides support to the colony; however, it apparently does not survive the colony's death for long since no dead skeletons were sampled here (personal observation) or reported previously. Some colonies of *H. finmarchica* showed no tissue on the older upper section where two ectobiotic species were found (sea anemone *S. nexilis* and a hydrozoan). The biodiversity in this older section was not consistently higher than elsewhere along the colony, which can be due to its small diameter and the smooth surface of the central axis, less favorable to settlement, as well as its susceptibility to erosion or to grazing predators [59].

Few ectobiotic species are reported on the living tissue of gorgonians and all are highly specialised symbionts [1]. Similarly rare ectobiotic species were identified on sea pens, none of which were found on the living tissues of *H. finmarchica* and only one on the soft tissues of *A. grandiflorum*: an egg mass of the eelpout *Lycodes esmarkii*. Ectobiotic species are probably rare because soft corals, including sea pens, produce toxic chemicals acting as antifouling agents [60]–[62]. A study on the shallow-water pennatulacean *Renilla octodentata* confirmed the negative effect of those agents on the settlement of barnacle [63]. Chemicals, if present, seem to have a limited impact on colonisation by endobiotic species, which represent 87.7% of the associates recorded here. The ability of endobionts to colonize sea pen tissues might be explained by the fact that most of them are parasitic and have developed adaptations to thwart their host's defenses [64]. Overall, 38.6% of the colonies of *H. finmarchica* harboured endobionts compared to 66.7% of *A. grandiflorum*, suggesting that the former may be better protected against infestations. The rachis of *H. finmarchica* produces a larger quantity of mucus than that of *A. grandiflorum* (personal observation), which might create a barrier against settlement and mitigate infestation.

While chemical deterrents produced by corals may influence colonisation by ectobionts and endobionts, they are also known to deter predators [61], [65]. Hence, corals may offer protective shelter to free-living associates. Keeping in mind that the sampling method (see below) likely underestimated the number of unattached faunal associates that derive shelter or food from sea pens, a clear difference in the number of free-living associates between the two sea pens was found. All 7 free-living associates were found on *A. grandiflorum* and only one (larvae of *Sebastes* spp.) on *H. finmarchica*. It is presumed that *A. grandiflorum* relies only on chemical defenses while *H. finmarchica* also harbours sclerites forming a calyce around the polyps (physical defense) [59]. However, the common observation of bare central axis in *H. finmarchica* suggests that this species is more often grazed than *A. grandiflorum*, possibly explaining why free-living associates might favour *A. grandiflorum*, which is predated by slow-moving species such as the sea star *Hippasteria phrygiana* [66]. Alternatively, the morphology of the two sea pens might explain this discrepancy. The elongated polyps of *A. grandiflorum* occur singly, while the polyp rows on *H. finmarchica* are fused at their base, forming ridges, as described by Williams [67]. Thus, *A. grandiflorum* is more “bushy” than *H. finmarchica*, which probably allows small invertebrates (e.g. shrimp larvae, copepods) and small vertebrates (e.g. fish larvae) to use *A. grandiflorum* for shelter and protection. The shallow-water sea pen *P. guerneyi* provides anchorage to various species against the tidal flow and a hiding place for small invertebrates, e.g. amphipods, caprellids and shrimps [29], emphasising the importance of sea pens as shelter and structural habitat. While *H. finmarchica* is a less likely shelter for free-living organisms, stomach contents of its ectobiont, the sea anemone *Stephanauge nexilis*, showed the presence of small invertebrates (free-living amphipods, copepods and halocypriods), suggesting their presence around colonies of *H. finmarchica*. The whip morphology of *H. finmarchica* may be less likely to retain small associates during sampling and lead to an underestimation of this type of association. Buhl-Mortensen and Mortensen [1] indicated that sampling of the associated species of deep-sea gorgonians by trawl led to the loss of most of the mobile crustaceans, which were sampled when using suction devices with a ROV. An additional challenge is that some free-living associates of sea pens are present only during a specific life stage and/or a specific season: three different species of shrimp larvae (*Acanthephyra pelagica*, *Pandalus montagui* and *Pasiphaea multidentata*) were found here in April/May (spring) exclusively. Previously, fish larvae of *Sebastes* spp. were also found on both species of sea pens in April and May, prompting the suggestion that sea pens act as essential fish habitat [3]. The additional presence of shrimp larvae underscores the importance of sea pens for the early life history of other species, including commercially harvested ones.

While transient free-living associates are important, the three most common associates found on both sea pens (*L. bouligandi* on *A. grandiflorum*, *S. nexilis* and undescribed Corallovexiidae on *H. finmarchica*) can be considered symbionts. *L. bouligandi* and the corallovexiid are endoparasites that spend most of their life history inside the polyp. While *A. grandiflorum* and *H. finmarchica* are sympatric species, their respective endoparasitic copepods are distinct. Lamippidae are adapted to their coral host [68] supporting the assumption that *L. bouligandi* is highly specific to *A. grandiflorum*. In contrast, Corallovexiidae are either monospecific or found in 2 or 3 closely related coral hosts [49], suggesting that the corallovexiid in *H. finmarchica* might yet be found in other sea pens. Parasitic copepods in *A. grandiflorum* predominated in the upper rachis section, whereas those in *H. finmarchica* occurred mostly in the middle section. This trend can be explained by the greater density and larger diameter of polyps in these sections, which correspond to older polyps [59], and thus provide greater opportunity for infestation.

Both copepods had an impact on the polyps they infested: a total absence of oocytes/spermatocysts suggesting an inhibition of gametogenesis in *H. finmarchica*, and ∼45% decrease in relative fecundity in *A. grandiflorum*. Parasitic copepods disrupt vitellogenesis (yolk deposition) either because they interfere with feeding or increase energy expenditure by the polyp (e.g. immune reaction). At the colony level, few polyps are infested, limiting the effect on total fecundity. Lamippidae were previously shown to increase mortality rates of sea pen hosts under stress (e.g. anoxic condition) despite their healthy appearance in optimal conditions [29]. Overall, copepods are the most common parasites identified in deep-sea octocorals [13]. Here, in addition to the two species discussed above, 7 individuals of an unidentified Lamippidae were recorded in the polyps of *H. finmarachica*. Furthermore, a copepod of the genus *Linaresia* was recently found in the polyps of a deep-sea gorgonian, *Paramuricea* sp., in the Northwest Atlantic [69].

The sea anemone *S. nexilis* found on *H. finmarchicus* is commonly reported from the Northwest Atlantic, between the Gulf of Mexico [70] and Labrador [71]. *S. nexilis* emerges as a facultative ectobiont with a low specificity for *H. finmarchica*. It is found attached to rocks, empty shells and sponges in the Gulf of Mexico [70]. The life history of this species is not known, but it can be hypothesised that it settles at the larval stage on the central axis of the sea pen and remains there due to the general absence of other suitable substrata where muddy seafloor dominates. Whether the absence of polyps around the sea anemones is a prerequisite to their settlement on sea pens, or an outcome of it, remains unclear. Some colonies of *H. finmarchica* exhibited a naked central axis without any visible ectobionts, suggesting that loss of soft tissue may precede colonization and supporting the assumption that the sea anemone is a commensal symbiont. On the other hand, a small number of sea anemones (probably newly settled) were observed to be closely surrounded by healthy tissues/polyps. Perhaps they initially settled on a small naked section of the colony and grew toward living tissues. It is not impossible that they are able to dislodge the polyps, which would correspond to a previously unreported case of parasitism.

The present study attempted to elucidate trophic relationships among sea pens and their principal associates. Previous work showed an increase of ∼3.8‰ in δ^15^N between prey and predator in polar and deep-sea environments [44]. Here, the endoparasitic copepods fell about half a trophic level above their sea pen hosts. Parasites are presumed to feed on a single source during a specific life stage [72], indicating that feeding on the host tissues should elicit a full trophic increase in δ^15^N, whereas feeding on the same food as the host should result in no difference between δ^15^N of parasite and host [73]. The intermediate values recorded here suggest that copepods might use a mixed strategy. This hypothesis is supported by the location of the parasitic copepods inside the polyps, which suggests that they can both feed directly on sea pen tissues and feed on items ingested by the polyp. Johnstone (1969) proposed a similar hypothesis for the diet of *Lamippe* sp. associated with the shallow-water *P. gurneyi* based on its location and on the observation of orange material in its digestive tract (the color of the sea pen's tissues). Our isotopic results also confirm that the sea anemone and both sea pens feed on sedimentary organic matter in addition to small pelagic invertebrates [74]. However, the sea anemone is potentially targeting different prey based on small invertebrates found in their gastro-vascular cavity, which were not observed in the sea pen polyps, suggesting that the sea anemone is not competing directly with its host for food.

Overall, sea pens appear to have a moderate number of associated species, as previously hypothesized [8]. Nevertheless, sea pens play important roles in the life history of their associates. Some, such as parasitic copepods spend most of, possibly all, their life in association with sea pens and depend on them to survive and reproduce. The presence of the sea anemone on *H. finmarchica* confirms that sea pens offer a suitable biogenic substrate for other species. Sea pens are also important for mobile species such as fishes and shrimps that use them transiently as shelter during early life stages indicating that sea pens can be considered as biogenic habitat. However, the seasonality in these associations as well as the distribution of the sea pens (patchy occurrence of sea pen fields) emphasizes the difficulty in gaining a comprehensive understanding of their role as biogenic habitats. The sampling method used in this study (trawl by-catch) does not allow precise determination of functional interactions with free-living associates or a quantitative analysis, as some associates might be lost during sampling. However, this method is advantageous by permitting a large spatial and temporal coverage, as well as a large sample size, allowing the identification of spatiotemporal patterns which would not be possible with other sampling methods (e.g. ROV). Importantly, co-occurrences were not investigated here; only close (physical) associations. Recent studies have shown that the sea star *Mediaster bairdi* is usually found in sea pen fields in the Northwest Atlantic [66], and that lobsters often occur in association with sea pens in Norway fjords [75] suggesting that the contribution of pennatulacean corals to deep-sea biodiversity has yet to be fully elucidated.

## Supporting Information

Table S1 **List of the sites analysed in 2006–2007. The symbol — indicates that no samples were analysed.**
(DOC)

Table S2 **List of sites analysed in 2009–2010.**
(DOC)

Table S3 **Results of DNA barcoding with GenBank accession numbers.**
(DOC)
