# Supplementary material for: Diversity, Distribution and Nature of Faunal Associations with Deep-Sea Pennatulacean Corals in the Northwest Atlantic
Source: PLoS One. 2014 Nov 4;9(11):e111519. doi: 10.1371/journal.pone.0111519 (PMC4219758; doi:10.1371/journal.pone.0111519)
Supplement: Table S1 — List of the sites analysed in 2006–2007. The symbol — indicates that no samples were analysed. (DOC) [file pone.0111519.s001.doc]

Table S1. List of the sites analysed in 2006-2007. The symbol -- indicates that no samples were analysed.

|  |  |  |  | **Number of colonies examined** | | **SMEY/SMY^b^ (individual colony^-1^)** | | | |
| --- | --- | --- | --- | --- | --- | --- | --- | --- | --- |
| **Site** | **Region^a^** | **Date** | **Depth (m)** | ***A. grandiflorum*** | ***H. finmarchica*** | ***A. grandiflorum*** | | ***H. finmarchica*** | |
| 1 | NNL | 26/01/2006 | 1167 | 2 | -- | 11 | 5.5 | -- | -- |
| 2 | NNL | 27/01/2006 | 918 | -- | 1 | -- | -- | 0 | 0 |
| 3 | NNL | 27/01/2006 | 1070 | 1 | -- | 6 | 6 | -- | -- |
| 4 | NNL | 30/01/2006 | 821 | 2 | -- | 11 | 5.5 | -- | -- |
| 5 | NNL | 12/02/2006 | 921 | 2 | -- | 6 | 3 | -- | -- |
| 6 | NNL | 15/03/2006 | 864 | 1 | -- | 1 | 1 | -- | -- |
| 7 | NNL | 31/03/2006 | 891 | 1 | -- | 1 | 1 | -- | -- |
| 8 | LB | 10/07/2006 | 1299 | 1 | -- | 0 | 0 | -- | -- |
| 9 | LB | 14/07/2006 | 837 | -- | 1 | -- | -- | 14 | 14 |
| 10 | LB | 27/7/2006 | 176 | 2 | -- | 21 | 21 | -- | -- |
| 11 | LB | 28/07/2006 | 552 | 3 | 5 | 7.7 | 7.7 | 3.3 | 2.6 |
| 12 | LB | 28/07/2006 | 620 | 5 | 2 | 13.6 | 13.6 | 4 | 4 |
| 13 | LB | 29/07/2006 | 452 | -- | 1 | -- | -- | 0 | 0 |
| 14 | LB | 30/07/2006 | 528 | -- | 1 | -- | -- | 1 | 1 |
| 15 | LB | 04/08/2006 | 563 | -- | 1 | -- | -- | 1 | 0.5 |
| 16 | LB | 04/08/2006 | 630 | -- | 2 | -- | -- | 1 | 0.5 |
| 17 | LB | 05/08/2006 | 559 | -- | 1 | -- | -- | 0 | 0 |
| 18 | LB | 08/08/2006 | 724 | -- | 1 | -- | -- | 4 | 4 |
| 19 | GB | 06/10/2006 | 618 | -- | 2 | -- | -- | 1 | 0.5 |
| 20 | LB | 07/10/2006 | 626 | 1 | -- | 53 | 53 | -- | -- |
| 21 | LB | 07/10/2006 | 466 | 1 | -- | 3 | 3 | -- | -- |
| 22 | LB | 09/10/2006 | 424 | -- | 1 | -- | -- | 0 | 0 |
| 23 | LB | 09/10/2006 | 803 | 1 | -- | 17 | 17 | -- | -- |
| 24 | LB | 15/10/2006 | 548 | 3 | -- | 17 | 17 | -- | -- |
| 25 | LB | 17/10/2006 | 895 | 1 | -- | 8 | 8 | -- | -- |
| 26 | LB | 02/11/2006 | 256 | -- | 1 | -- | -- | 0 | 0 |
| 27 | LB | 04/11/2006 | 1071 | 1 | 0 | 1 | 1 | -- | -- |
| 28 | FC | 24/11/2006 | 1018 | 1 | -- | 1 | 1 | -- | -- |
| 29 | FC | 27/11/2006 | 958 | 2 | -- | 6 | 6 | -- | -- |
| 30 | FC | 28/11/2006 | 1073 | -- | 2 | -- | -- | 0 | 0 |
| 31 | FC | 28/11/2006 | 788 | -- | 1 | -- | -- | 1 | 1 |
| 32 | FC | 28/11/2006 | 1134 | 4 | -- | 3.3 | 2.5 | -- | -- |
| 33 | FC | 28/11/2006 | 1125 | 20 | 4 | 7.8 | 7.4 | 1.5 | 0.8 |
| 34 | FC | 30/11/2006 | 780 | -- | 2 | -- | -- | 0 | 0 |
| 35 | FC | 01/12/2006 | 1161 | -- | 3 | -- | -- | 0 | 0 |
| 36 | FC | 01/12/2006 | 1186 | 2 | -- | 9.5 | 9.5 | -- | -- |
| 37 | FC | 01/12/2006 | 1036 | 3 | -- | 11 | 11 | -- | -- |
| 38 | FC | 02/12/2006 | 978 | 1 | -- | 3 | 3 | -- | -- |
| 39 | LC | 14/04/2007 | 435 | 8 | -- | 3.3 | 1.3 | -- | -- |
| 40 | LC | 15/04/2007 | 457 | 3 | -- | 38 | 12.7 | -- | -- |
| 41 | LC | 15/04/2007 | 456 | 1 | -- | 9 | 9 | -- | -- |
| 42 | LC | 15/04/2007 | 428 | 4 | -- | 16.7 | 12.5 | -- | -- |
| 43 | LC | 16/04/2007 | 457 | 3 | -- | 13.3 | 13.3 | -- | -- |
| 44 | LC | 16/04/2007 | 352 | 6 | -- | 5.8 | 4.8 | -- | -- |
| 45 | GB | 20/04/2007 | 377 | -- | 2 | -- | -- | 0 | 0 |
| 46 | LC | 28/04/2007 | 370 | 3 | 3 | 17 | 9.3 | 13.3 | 13.3 |
| 47 | LC | 29/04/2007 | 366 | -- | 4 | -- | -- | 6 | 4.5 |
| 48 | LC | 29/04/2007 | 462 | 2 | -- | 5 | 5 | -- | -- |
| 49 | LC | 30/04/2007 | 389 | -- | 2 | -- | -- | 8 | 8 |
| 50 | GB | 30/04/2007 | 98 | 1 | -- | 1 | 1 | -- | -- |
| 51 | LC | 30/04/2007 | 422 | 4 | 2 | 21.5 | 21.5 | 5.5 | 5.5 |
| 52 | GB | 04/05/2007 | 575 | -- | 4 | -- | -- | 1 | 0.3 |
| 53 | GB | 17/05/2007 | 425 | -- | 1 | -- | -- | 6 | 6 |
| 54 | GB | 17/05/2007 | 214 | 1 | -- | 0 | 0 | -- | -- |
| 55 | GB | 17/05/2007 | 597 | 3 | 2 | 3.5 | 2.3 | 1 | 0.5 |
| 56 | GB | 17/05/2007 | 600 | 4 | -- | 2.8 | 2.8 | -- | -- |
| 57 | GB | 18/05/2007 | 625 | -- | 1 | -- | -- | 0 | 0 |
| 58 | FC | 18/06/2007 | 658 | 2 | -- | 0 | 0 | -- | -- |
| 59 | FC | 08/07/2007 | 701 | 1 | -- | 0 | 0 | -- | -- |
| 60 | LB | 17/07/2007 | 881 | 3 | -- | 9 | 9 | -- | -- |
| 61 | LB | 18/07/2007 | 871 | 3 | -- | 8.3 | 8.3 | -- | -- |
| 62 | LB | 20/07/2007 | 883 | 2 | -- | 30 | 30 | -- | -- |
| 63 | LB | 21/07/2007 | 874 | 2 | -- | 31 | 31 | -- | -- |
| 64 | FC | 27/07/2007 | 267 | 1 | -- | 1 | 1 | -- | -- |
| 65 | GB | 07/10/2007 | 773 | -- | 5 | -- | -- | 2 | 0.4 |
| 66 | GB | 08/10/2007 | 1034 | -- | 1 | -- | -- | 0 | 0 |
| 67 | GB | 08/10/2007 | 823 | -- | 4 | -- | -- | 1 | 0.3 |
| 68 | GB | 08/10/2007 | 1094 | -- | 1 | -- | -- | 0 | 0 |
| 69 | GB | 08/10/2007 | 796 | -- | 1 | -- | -- | 0 | 0 |
| 70 | GB | 08/10/2007 | 1333 | 2 | 1 | 1 | 0.5 | 2 | 2 |
| 71 | GB | 08/10/2007 | 824 | 3 | -- | 3.7 | 3.7 | -- | -- |
| 72 | GB | 09/10/2007 | 1130 | 2 | -- | 1 | 0.5 | -- | -- |
| 73 | GB | 10/10/2007 | 1347 | 1 | -- | 5 | 5 | -- | -- |
| 74 | GB | 10/10/2007 | 836 | 2 | -- | 6 | 6 | -- | -- |
| 75 | GB | 10/10/2007 | 934 | 6 | -- | 3.6 | 3 | -- | -- |
| 76 | LB | 16/10/2007 | 986 | 1 | -- | 1 | 1 | -- | -- |
| 77 | FC | 18/10/2007 | 1036 | -- | 2 | -- | -- | 4 | 2 |
| 78 | FC | 18/10/2007 | 1151 | -- | 1 | -- | -- | 0 | 0 |
| 79 | FC | 18/10/2007 | 768 | -- | 1 | -- | -- | 5 | 5 |
| 80 | FC | 18/10/2007 | 1061 | -- | 1 | -- | -- | 0 | 0 |
| 81 | FC | 18/10/2007 | 896 | 6 | -- | 4 | 0.7 | -- | -- |
| 82 | FC | 18/10/2007 | 1208 | 4 | 3 | 4 | 3 | 0 | 0 |
| 83 | FC | 19/10/2007 | 743 | 2 | -- | 5 | 2.5 | -- | -- |
| 84 | FC | 19/10/2007 | 743 | 2 | -- | 1 | 0.5 | -- | -- |
| 85 | GB | 28/10/2007 | 599 | 3 | 1 | 5 | 3.3 | 4 | 4 |
| 86 | FC | 29/10/2007 | 992 | -- | 4 | -- | -- | 2 | 0.5 |
| 87 | GB | 29/10/2007 | 579 | 1 | 2 | 0 | 0 | 3 | 1.5 |
| 88 | GB | 30/10/2007 | 593 | 2 | 2 | 8 | 4 | 1.5 | 1.5 |
| 89 | GB | 30/10/2007 | 623 | 4 | 3 | 12.5 | 12.5 | 2.7 | 2.7 |
| 90 | LB | 14/11/2007 | 1041 | -- | 1 | -- | -- | 0 | 0 |
| 91 | LB | 16/11/2007 | 1039 | 1 | -- | 22 | 22 | -- | -- |
| 92 | FC | 25/11/2007 | 651 | 1 | -- | 7 | 7 | -- | -- |
| 93 | FC | 26/11/2007 | 273 | 2 | -- | 20 | 10 | -- | -- |
| 94 | FC | 29/11/2007 | 810 | 2 | 1 | 1 | 0.5 | 0 | 0 |
| 95 | NNL | 08/12/2007 | 656 | 1 | -- | 0 | 0 | -- | -- |
| 96 | NNL | 14/12/2007 | 851 | 1 | -- | 0 | 0 | -- | -- |
| 97 | NNL | 14/12/2007 | 1285 | 1 | -- | 5 | 5 | -- | -- |

^a^ LC: Laurentian Channel, GB: Grand Banks, FC: Flemish Cap, NNL: North Newfoundland, LB: Labrador

^b^ SMEY: Site Mean Exact Yield, SMY: Site Mean Yield
