# Supplementary material for: Diversity, Distribution and Nature of Faunal Associations with Deep-Sea Pennatulacean Corals in the Northwest Atlantic
Source: PLoS One. 2014 Nov 4;9(11):e111519. doi: 10.1371/journal.pone.0111519 (PMC4219758; doi:10.1371/journal.pone.0111519)
Supplement: Table S2 — List of sites analysed in 2009–2010. (DOC) [file pone.0111519.s002.doc]

Table S2. List of sites analysed in 2009-2010.

|  |  |  |  | **Number of colonies examined** | |
| --- | --- | --- | --- | --- | --- |
| **Site** | **Region*** | **Date** | **Depth (m)** | ***A. grandiflorum*** | ***H. finmarchica*** |
| 98 | LC | 08/05/2008 | 301 | 1 | 0 |
| 99 | LC | 09/05/2008 | 462 | 1 | 0 |
| 100 | GB | 11/04/2009 | 494 | 2 | 0 |
| 101 | GB | 11/04/2009 | 428 | 1 | 0 |
| 102 | LC | 17/04/2009 | 318 | 4 | 0 |
| 103 | LC | 18/04/2009 | 437 | 2 | 0 |
| 104 | LC | 18/04/2009 | 428 | 1 | 0 |
| 105 | LC | 19/04/2009 | 453 | 2 | 0 |
| 106 | LC | 19/04/2009 | 299 | 1 | 0 |
| 107 | LC | 19/04/2009 | 317 | 3 | 0 |
| 108 | LC | 19/04/2009 | 445 | 6 | 0 |
| 109 | LC | 29/04/2009 | 451 | 3 | 0 |
| 110 | LC | 30/04/2009 | 488 | 1 | 0 |
| 111 | LC | 30/04/2009 | 348 | 3 | 0 |
| 112 | LC | 03/05/2009 | 265 | 1 | 0 |
| 113 | LC | 10/05/2009 | 404 | 3 | 0 |
| 114 | LC | 11/05/2009 | 404 | 1 | 0 |
| 115 | LC | 11/05/2009 | 422 | 3 | 2 |
| 116 | LC | 12/05/2009 | 410 | 0 | 1 |
| 117 | GB | 24/05/2009 | 337 | 2 | 2 |
| 118 | GB | 24/05/2009 | 603 | 3 | 4 |
| 119 | GB | 26/05/2009 | 596 | 3 | 1 |
| 120 | LC | 14/04/2010 | 384 | 0 | 2 |
| 121 | LC | 15/04/2010 | 314 | 2 | 0 |
| 122 | LC | 24/04/2010 | 456 | 1 | 0 |
| 123 | LC | 25/04/2010 | 449 | 1 | 0 |
| 124 | LC | 26/04/2010 | 455 | 1 | 0 |
| 125 | LC | 26/04/2010 | 468 | 1 | 0 |
| 126 | LC | 26/04/2010 | 433 | 3 | 0 |
| 127 | LC | 27/04/2010 | 447 | 3 | 0 |
| 128 | LC | 11/05/2010 | 673 | 1 | 0 |

*LC: Laurentian Channel, GB: Grand Banks, FC: Flemish Cap, NNL: North Newfoundland, LB: Labrador
