# Supplementary material for: Diversity, Distribution and Nature of Faunal Associations with Deep-Sea Pennatulacean Corals in the Northwest Atlantic
Source: PLoS One. 2014 Nov 4;9(11):e111519. doi: 10.1371/journal.pone.0111519 (PMC4219758; doi:10.1371/journal.pone.0111519)
Supplement: Table S3 — Results of DNA barcoding with GenBank accession numbers. (DOC) [file pone.0111519.s003.doc]

Table S3: Results of DNA barcoding with GenBank accession numbers.

| **Species/taxa** | **GenBank number** |
| --- | --- |
| *Acanthephyra pelagica* | KF930998 |
| Halocyprida | KF931023 |
| *Lamippe bouligandi* | KF931032 |
| *Lamippe bouligandi* | KF931014 |
| *Lamippe bouligandi* | KF931015 |
| *Lamippe bouligandi* | KF931029 |
| *Lamippe bouligandi* | KF931025 |
| *Lamippe bouligandi* | KF931016 |
| *Lamippe bouligandi* | KF931030 |
| *Lamippe bouligandi* | KF931028 |
| *Lamippe bouligandi* | KF931027 |
| *Lamippe bouligandi* | KF931026 |
| *Lamippe bouligandi* | KF931031 |
| *Pandalus montagui* | KF931035 |
| *Pandalus montagui* | KF931033 |
| *Pandalus montagui* | KF931034 |
| *Paraeuchaeta norvegica* | KF931022 |
| *Pasiphaea multidentata* | KF931036 |
| *Pseudosagitta maxima* | KF931010 |
| *Pseudosagitta maxima* | KF931011 |
| *Pseudosagitta maxima* | KF931009 |
| *Sagitta maxima* | KF931008 |
| *Stephanauge nexilis* | KF931038 |
| *Stephanauge nexilis* | KF931041 |
| *Stephanauge nexilis* | KF931042 |
| *Stephanauge nexilis* | KF931045 |
| *Stephanauge nexilis* | KF931044 |
| *Stephanauge nexilis* | KF931043 |
| *Stephanauge nexilis* | KF931046 |
| *Stephanauge nexilis* | KF931047 |
| *Stephanauge nexilis* | KF931040 |
| *Stephanauge nexilis* | KF931039 |
| *Stephanauge nexilis* | KF931037 |
| Undescribed Corallovexiidae | KF931017 |
| Undescribed Corallovexiidae | KF931013 |
| Unidentified Amphipoda | KF931003 |
| Unidentified Amphipoda | KF931000 |
| Unidentified Amphipoda | KF931001 |
| Unidentified Amphipoda | KF931002 |
| Unidentified Copepoda | KF931007 |
| Unidentified Copepoda | KF931006 |
| Unidentified Copepoda | KF930999 |
| Unidentified Copepoda | KF931005 |
| Unidentified Copepoda | KF931018 |
| Unidentified Copepoda | KF931019 |
| Unidentified Copepoda | KF931020 |
| Unidentified Copepoda | KF931021 |
| Unidentified Hydrozoa | KF931024 |
| Unidentified sp. 6 | KF931012 |
